# Analysis of Antibodies Induced after SARS-CoV-2 Vaccination Using Antigen Coded Bead Array Luminex Technology

**DOI:** 10.3390/vaccines11020442

**Published:** 2023-02-15

**Authors:** Zixuan Song, Qizhi Luo, Ling Wan, Quan Zhu, Rongjiao Liu, Xiangli Yin, Xiaofang Lu, Leiyan Wei, Zhiqing Xiang, Yizhou Zou

**Affiliations:** Department of Immunology, School of Basic Medical of Central South University, Changsha 410083, China

**Keywords:** SARS-CoV-2, variants of concern, CoronaVac, BBIBP-CorV, ZF2001, IgG assay

## Abstract

***Objectives***. Since the outbreak of SARS-CoV-2 in late 2019, nearly 12.2 billion doses of the COVID-19 vaccine have been administered worldwide; however, the humoral immune responses induced by different types of vaccines are yet to be fully validated. ***Methods***. We analyzed antibody levels in 100 serum samples after vaccination with different types of COVID-19 vaccines and their reactivity against the RBD antigen of Delta and Omicron variants using a bead-based microarray. ***Results***. Elevated levels of anti-wild-type (WT)-RBD IgG and anti-WT-NP IgG were detected in participants who received two doses of the inactivated vaccines (CoronaVac or BBIBP-CorV) and three doses of the recombinant spike protein vaccine (ZF2001), indicating that antibody responses to SARS-CoV-2 were generated regardless of the vaccine administered. We found highly correlated levels of serum anti-RBD IgG and anti-NP IgG (r = 0.432, *p* < 0.001). We observed that the antibodies produced in vivo after COVID-19 vaccination still reacted with variants of SARS-CoV-2 (*p* < 0.0001). ***Conclusions***. Our results show that high levels of specific antibodies can be produced after completion of COVID-19 vaccination (two doses of the inactivated vaccines or three doses of ZF2001), with some degree of cross-reactivity to the RBD antigen of Delta and Omicron variants, and provide an accessible and practical experimental method for post-vaccination antibody detection.

## 1. Introduction

Since December 2019, severe acute respiratory syndrome coronavirus 2 (SARS-CoV-2) has been spreading rapidly worldwide, leading to an ongoing pandemic of coronavirus disease 2019 (COVID-19) with approximately 670 million infections and 6.83 million deaths as of 31 January 2023 (https://coronavirus.jhu.edu/map.html (accessed on 10 January 2023)).

To date, 11 vaccines have been included in the World Health Organization emergency use list, mainly including: (1) Two mRNA-based vaccines expressing full spike (S) glycoprotein, BNT162b2 (by Pfizer) and mRNA-1273 (by Moderna), with clinical trial data showing that BNT162b2 and mRNA-1273 were 95% [1] and 94.1% [2] effective against COVID-19, respectively; (2) Chimpanzee adenovirus vector vaccine encoding the full S glycoprotein, named ChAdOx1 nCoV-19 (by AstraZeneca), the efficiency rate of which was shown to be 62.1% [3]; (3) The human adenovirus vector vaccine encoding the full S glycoprotein is named Ad26.CoV2.S (by Johnson & Johnson), which provides 66.9% protection against moderate-to-severe COVID-19 [4]; (4) Two inactivated whole-coronavirus vaccines, CoronaVac (by Sinovac) and BBIBP-CorV (by SinoPharm), with efficiency rates of 50.7% [5] and 72.8% [6], respectively. Nine SARS-CoV-2 vaccines have been approved by China’s State Drug Administration for conditional listing or emergency use, including CoronaVac, BBIBP-CorV, the adenovirus type 5 vector recombinant COVID-19 vaccine Ad5-nCoV-S CONVIDECIA (by CanSino), and the recombinant spike protein vaccine ZF2001 (by Anhui Zhifei Longcom). The data from a phase 3 clinical trial of three doses of ZF2001 showed that the vaccine had 75.7% protection [7].

Coronaviruses use homotrimeric spike glycoproteins on the envelope to bind to the cellular receptor angiotensin converting enzyme-2 (ACE-2), leading to fusion of the cellular and viral membranes for entry into the cell [8,9,10]. Therefore, the titer of anti-receptor-binding-domain (RBD) IgG and neutralization titer are highly correlative. Hence, anti-RBD IgG is typically used to evaluate neutralizing antibodies after vaccination.

The emergence of variants of concern (VOCs) poses a significant challenge to the effective protection provided by vaccine induction [11,12,13,14,15,16]. The peak of the epidemic in late 2021 was attributed to the Omicron variant (B.1.1.529), which contains 15 amino acid mutations in the RBD [17] and has a significantly enhanced immune escape capacity [18], making it a consistently dominant variant for causing large number of infections worldwide [19]. Thus, it is crucial to investigate whether vaccine-induced antibody production induces an immune response against VOCs.

Luminex microspheres contain three types of fluorescent immunofluorescence, and 100 different kinds of microspheres can be distinguished by different ratios of fluorescence. Each microsphere can be used to detect a different protein or gene. As a result, up to 100 proteins or genes can be detected simultaneously using microsphere technology. This technique uses fluorescent-encoded microspheres covalent cross-linked protein to bind to the target molecule, add fluorescein-labeled detection antibody, and then identify single microspheres by laser scanning fluorescence coding and measure fluorescence intensity to determine the concentration of the measured molecule. Due to the advantages of fast detection results and high accuracy, it has been shown that it can be used for cytokine detection [20] and HLA typing before transplantation [21,22].

Our study aims to provide some technical supports for the establishment of SARS-CoV-2 protein microarrays in the future through molecular cloning, protein expression and Luminex technology. Protein microarrays can characterize proteome-wide antibody responses in high-throughput format, providing a more systematic description of the IgG antibody repertoire in vivo after COVID-19 vaccination.

The application of a bead-based microarray system allows the labeling of a single antigen molecule in colored microspheres and the assembly of different antigenic protein molecules of coronavirus (S protein, N protein, and other variant antigenic proteins) for the quantitative detection of one to dozen specific anti-coronavirus antibodies that may be present in serum samples. Thus, herein, we applied the Luminex system to probe humoral immune responses in participants receiving vaccination.

## 2. Methods

### 2.1. Study Participants

This study enrolled 100 volunteers between April and November 2021, of which 14 who received CoronaVac were followed up. Ninety-seven percent of the participants were university students and staff. The exclusion criteria were as follows: patients with (1) documented SARS-CoV-2 infection, (2) severe blood and other serious diseases, and (3) COVID-19 symptoms such as sore throat, fever, and cough. Informed consent was obtained from all individual participants included in the study.

### 2.2. Antigens and Antibodies

We ordered the recombinant nucleocapsid protein (NP) and divided the intact NP antigen protein into three segments: NP1 (Cat#: Pr40001, ProMab), NP2 (Cat#: Pr40002, ProMab), and NP3 (Cat#: Pr40003, ProMab). We purchased SARS-CoV-2 wild-type (WT)-RBD antigen protein (Cat#: Pr40004, ProMab) from Hunan Yuantai Biotechnology Co. and Omicron-RBD antigen protein (Cat#: 40592-V08H121, Sino Biological Inc., Beijing, China) and SARS-CoV-2 spike mouse monoclonal antibody (Cat#: 40592-MM117, Sino Biological Inc.) from Beijing Sino Biological Technology Co. Ltd. (Beijing, China).

### 2.3. Construction of the Recombinant Expression Vectors

The designed pFlag-CMV5.1-WT-RBD nucleotide sequence was synthesized by Beijing Tsingke Biotechnology Co. Ltd. Two pairs of primers: (1) HindIII-Delta-F (5′-CCCAAGCTTGCCACCATGGGCCTGGGGCCTGTG-3′) and R-Delta-F(5′-GATGTCTCTTTCGAAGGGTTTCAGGTTGCTCTTTCTGAACAGTCTGTACCGGTAGTTGTAGTTGCC-3′) and (2) F-Delta-R (5′-CCTTCGAAAGAGACATCAGCACCGAGATTTACCAGGCCGGAAGCAAACCATGCAACGGAGTG-3′) and R-Delta-XbaI (5′-GCTCTAGATCAGTGGTGGTGGTGATG-3′) were used to amplify Arg319–Asp491 and Leu484–Phe541 of the Delta-RBD coding sequences, respectively. Polymerase chain reaction ligation of these two fragments was performed using the two-end primers HindIII-Delta-F and R-Delta-XbaI. The newly amplified Delta-RBD fragment was ligated with T4 ligase (Cat#: 01001207, Thermo Scientific, Waltham, MA, USA) to the eukaryotic expression vector pFlag-CMV5.1 with HindIII (Cat#: R3104V, NEB) and XbaI (Cat#: 01007777, Thermo Scientific) between the two digestion sites. After transfecting the synthetic plasmid and ligation product into competent *Escherichia coli* DH5α cells (Cat#: G6016–20, ANGYUBIO), the target colonies were amplified, and the plasmids were extracted for sequencing verification. Plasmids with the correct sequences were selected for the next step of the experiment.

### 2.4. Expression and Purification of the Recombinant Protein

The synthetic plasmid pFlag-CMV5.1-WT-RBD and the ligation product pFlag-CMV5.1-Delta-RBD were mixed with the transfection reagent polyethyleneimine (Cat#: 23966, Polysciences Inc., Warrington, PA, USA) at a ratio of 1:3 for 15 min at room temperature, followed by transfection of HEK293 cell suspensions at a final concentration of 1 µg/mL of the mixture. HEK293 cell suspensions were obtained from our laboratory stock and cultured in dedicated serum-free SMM 293-TII medium (Cat#: M293TII, Sino Biological Inc.) at 37 °C in a 5% CO_2_ incubator. Cell supernatants were collected on day 3 after transfection. Subsequently, all cell supernatants were poured into the dialysis bag and completely immersed in dialysis buffer (pH 7.4) for dialysis at 4 °C. The dialysis solution was changed every 8 h until the supernatants became clear. Cell supernatants that were dialyzed for clarity were purified by nickel column affinity chromatography (Cat#: AA0052, Bestchrom, Shanghai, China) (using phosphate buffer containing 0.5 M imidazole as the eluent and phosphate buffer containing 40 mM imidazole as the buffer) and concentrated on an ultrafiltration column (Cat#: R1KB25424, Amicon^®^ Ultra-4) at −80 °C for storage.

### 2.5. Coomassie Brilliant Blue Staining

The lab-produced recombinant proteins WT-RBD and Delta-RBD were concentrated with 5% sodium dodecyl sulfate (SDS)-polyacrylamide gel electrophoresis (PAGE) and then separated by 12% SDS-PAGE. After electrophoresis, the proteins were stained with Komas Brilliant Blue dye (Cat#: R-250, SIGMA, St. Louis, MO, USA) for 30 min at room temperature and decolorized overnight with a decolorizing solution until clear bands appeared.

### 2.6. Western Blot Analysis

After the protein electrophoretic separation, the proteins were transferred to polyvinylidene difluoride membranes (Cat#: PVH00010, Immobilon-P, Darmstadt, Germany). After blocking with 5% skim milk for 1 h at 37 °C, anti-6 × his primary antibody was added and incubated overnight at 4 °C, and washed three times with 0.1% phosphate buffered solution (PBS)-Tween (PBST). Next, the membranes were incubated at 37 °C for 45 min with horseradish peroxidase-coupled anti-mouse IgG Fc secondary antibody (Cat#: 115–035–071, Jackson Immuno-Research Labs, West Grove, PA, USA), followed by three washes with 0.1% PBST. Finally, the ECL chemiluminescence detection reagent (Cat#: M507M01, Absin, Shanghai, China) was added to the membrane, and the results were observed using a luminometer (Tanon^®^ 5200Multi).

### 2.7. Luminex Bead-Based Immunoassay

According to the instructions of Bio-Plex^®^ 200 System (BIO-RAD, Hercules, CA, USA), we coupled 12.5 μg (the maximum amount of antigenic protein) of commercial NP1, NP2, NP3, WT-RBD, Omicron-RBD antigen protein, purified WT-RBD, and Delta-RBD antigen protein onto 2.5 × 10^6^ microbeads (i.e., 200 μL of 1.25 × 10^7^/mL microbead stock solution) for IgG detection. After centrifugation, the microbeads were resuspended with 0.1 M NaH_2_PO_4_ (pH 6.2). 50 mg/mL of Sulfo-NHS (Cat#: JH126614, Thermo) and EDC (Cat#: JL127870, Thermo) were prepared, and 10 μL of each was added to the microbead suspension, shaken for 20 min in the dark. After shaking, the activated microbeads were centrifuged and resuspended in 50 mM MES (pH 5.0). After repeating this operation twice, 200 μL of the solution containing 12.5 μg of antigenic protein prepared with activation buffer was added and shaken for 2 h at room temperature in the dark. The beads were centrifuged, washed twice with 0.05% PBST, and resuspended in 50% PBS-goat serum (Cat#: SLBQ0738V, SIGMA) overnight at 4 °C. After blocking, the microbeads were washed twice, resuspended in 10% PBS-goat serum, and stored in the dark at 4 °C.

We followed the protocol of previous investigators [21]. A mixture of 2 μL serum and 58 μL 10% PBS-goat serum was incubated for 30 min (i.e., the serum was diluted at 1:30), while 1 μL each of microbeads was added separately. After washing with 0.05% PBST for three times, PE-labeled anti-human IgG secondary antibody (Cat#: LMCJ120200, Gen-Probe, Transplant Diagnostics, Inc., West Avenue Stamford, CT, USA) was added and incubated in the dark for 30 min and then washed with 0.05% PBST three times. The mixture was added in a 96-well plate, and reactions were read using a Bio-Plex^®^ 200 System analyze. The results are expressed as median fluorescence intensity (MFI).

### 2.8. Statistical Analysis

Statistical analysis was performed using SPSS 26.0 and GraphPad Prism 8 software. Comparisons of class values between two or more groups were performed using a two-sided chi-squared test or Fisher’s method. After logarithmic MFI values were taken, a Kolmogorov-Smirnov test showed a normal distribution, and then statistical analysis was conducted. Quantitative data analyzed using independent sample *t*-tests, and pairwise comparisons were performed using paired *t*-tests. Multiple sample means were compared by analysis of variance (ANOVA), the LSD (Least-Significant Difference) test (two-by-two), and Dunnett’s test (two-by-two in a randomized group design with mouse IgG as the control group). Correlation analysis was performed using Pearson’s test, and one-way linear regression analysis was used to explore the factors affecting antibody levels. Data that were not normally distributed were analyzed by the Wilcoxon test. Statistical significance was set at *p* < 0.05.

## 3. Results

### 3.1. Demographic Characteristics of the Study Participants

The 100 volunteers included in this study received different types of COVID-19 vaccines between May 2021 and October 2021. The median age and body mass index (BMI) of the study participants (*n* = 100; male 42.0% and female 58.0%) was 20 years [interquartile range (IQR), 19–23] and 21.51 (IQR 19.31–24.61), respectively. Of the total participants, 30.0%, 24.0%, 2.00%, and 41.0% had blood type A, B, AB and O, respectively. The median time between the two doses of vaccination was 30 days (IQR 23–33), and the median time between the completion of the last dose of vaccination and the collection of blood samples was 76 days (IQR 30.5–109.25) (Table S1). Of the total participants, 55, 32, 8 and 5 received two doses of CoronaVac, two doses of BBIBP-CorV, three doses of ZF2001, and two doses of a mixed CoronaVac and BBIBP-CorV inactivated vaccine, respectively.

Based on the different distribution characteristics of the baseline information, we analyzed whether some variables have statistical differences in different vaccination groups, including sex (*p* = 0.424), blood type (*p* = 0.751), age (*p* = 0.257), BMI (*p* = 0.678), days between two doses of vaccination (*p* = 0.735), and days between completion of vaccination and collection of blood samples (*p* = 0.298). The analysis result showed that there were no statistical differences in the basic information and demographic composition of the 100 volunteers (Table 1), which allowed further testing and analysis of the collected peripheral blood samples.

### 3.2. Generation of the ACE-2 Receptor-Binding Domain Soluble Antigenic Protein from Wild-Type and Delta Variant Coronaviruses

**Figure 1:**
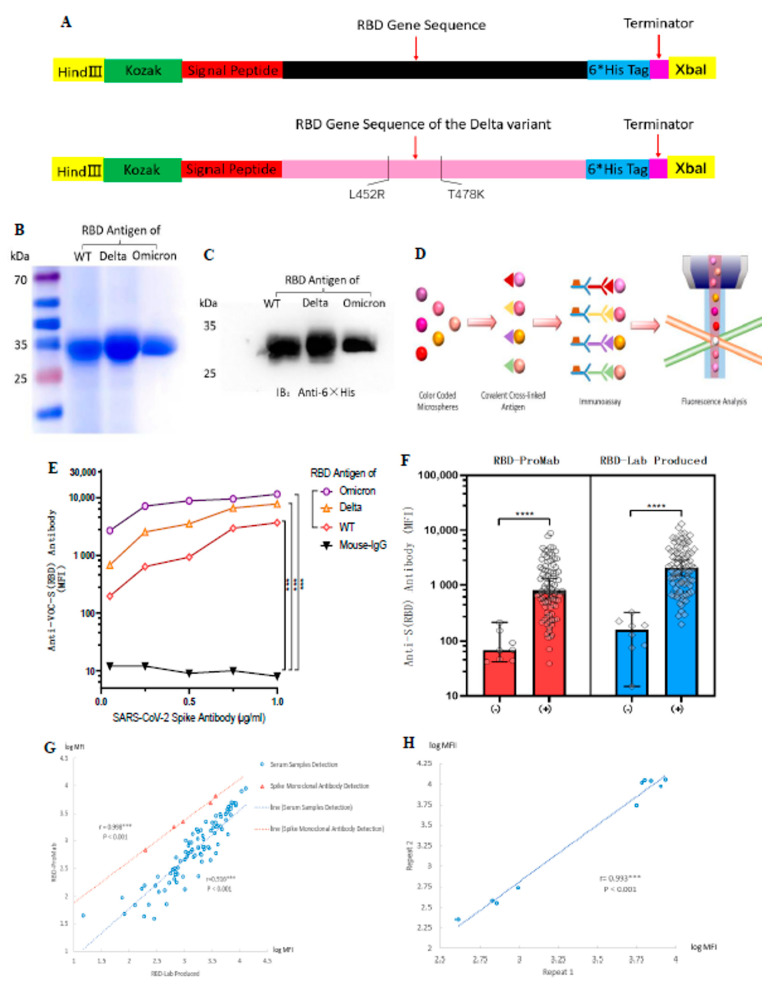
Design and generation of the ACE-2 receptor binding domain (RBD) soluble antigenic protein of SARS-CoV-2 and its VOCs. Luminex technology was used to develop a method to detect antibody levels after vaccination. (**A**) Cloning of the RBD gene. The RBD protein of the Delta variant had only two amino acid mutations. (**B**,**C**) Expression and purification of soluble antigenic protein RBD. (**D**) Schematic illustration of Luminex technology. (**E**) SARS-CoV-2 spike monoclonal antibody to validate WT-RBD and Delta-RBD antigen protein functions. The mean fluorescence intensity (MFI) values represent an indicator for the assessment of antigen-antibody binding. Mouse IgG was used as a negative control group for statistical analysis using Dunnett’s test. (**F**) Comparison of RBD-ProMab with RBD-Lab produced in negative (*n* = 8) and positive sera (*n* = 85). MFI values were logged, and the means were compared using independent sample *t*-test. (**G**) Correlation of RBD-ProMab and RBD-Lab produced antigenic protein function was analyzed by applying serum samples (*n* = 93) and monoclonal antibodies. (**H**) Correlation of anti-RBD IgG signal strength in two replicates of the same serum sample (*n* = 10). (**G**,**H**) MFI values were logged, and correlation analysis was performed using Pearson test. *** *p* < 0.001 and **** *p* < 0.0001.

To comprehensively assess anti-SARS-CoV-2 antibody production in serum, we prepared an RBD of the spike protein from wild-type and Delta variant coronaviruses as a target antigenic protein for detecting antibody levels in serum. Based on the PubMed reference genome for SARS-CoV-2 (GenBank No. MN908947.3) and reference SARS-CoV-2 spike protein functional partition on the UniProt website (https://www.uniprot.org/uniprot/P0DTC2 (accessed on 10 January 2023)), we determined the RBD protein amino acid sequence Arg319–Phe541. The Delta variant of the RBD protein target sequence was designed in the same way, with only two amino acid mutations, L452R and T478K (Figure 1A). The purified proteins were stained with Komas Brilliant Blue dye (Figure 1B) and analyzed by western blotting (Figure 1C). The results showed that the purified WT and Delta variant of the RBD protein had clear bands, indicating good purity of the target protein and consistent with the expected size of 27 kDa. The purchased Omicron-RBD (B.1.1.529) antigen protein was used as a control.

To evaluate the biological activity of the purified RBD protein, we verified its function using a mouse monoclonal antibody against the SARS-CoV-2 spike protein. At the same time, we hoped to measure antibody levels in serum by using a standard curve determined by gradient dilution of monoclonal antibodies, as in cytokine assays. RBD-Lab produced and RBD-ProMab antigen proteins were conjugated to Luminex color microspheres of different colors and incubated with gradient-diluted monoclonal antibodies. Afterward, PE-labeled secondary antibodies were added and their fluorescence intensity was detected with a Luminex instrument to represent their serum antibody levels (Figure 1D). Results showed that both lab-produced WT-RBD and Delta-RBD antigen proteins showed strong positive results compared with the negative control (mouse IgG) (*p* < 0.001) (Figure 1E). In contrast, the purchased Omicron-RBD protein generally had higher MFI.

The aberrant recognition by mouse mAb was likely due to its specific epitope that was better presented on Omicron variant. SARS-CoV-2 spike mouse monoclonal antibody from Sino Biologicals used to test RBD proteins is marketed as Omicron reactive by the manufacturer. The Sino Biologicals website indicates that this mAb has cross-reactivity with Delta and WT-RBD, but it is not clear if it was similar to Omicron.

We then compared the biological function of RBD-ProMab with RBD-Lab, produced by negative and positive sera. Both the RBD-ProMab- and RBD-Lab-produced proteins reacted with antibodies in vaccinated sera, and no significant antigen–antibody reactions were observed in the negative sera (Figure 1F). Although the RBD-Lab produced antigen protein was functionally similar to the purchased WT-RBD protein, there was a trend toward slightly higher MFI values for the lab-produced protein in both COVID-19 negative and COVID-19 positive sera. This may be due to the fact that lab-produced proteins still have a small amount of impurities. The results of the Komas Brilliant Blue dye indicated that the background of the laboratory-produced antigenic protein is still slightly bluish compared to the commercial protein lane, which may have a limited effect on the specificity of the experimental results. We calculated the Pearson correlation coefficient between RBD-ProMab and RBD-Lab produced using serum sample detection (r = 0.916, *p* < 0.001) and monoclonal antibody detection with a concentration gradient design (r = 0.998, *p* < 0.001) (Figure 1G). The results revealed no statistical difference in function between the purchased commercial and the lab-produced WT-RBD protein, and the latter could be used as the target antigen protein for detecting antibody levels.

To determine experimental reproducibility of serum antibody levels using antigen coded bead array, we randomly selected 10 serum samples for two repeat tests. The Pearson correlation coefficient of anti-RBD IgG was 0.993 (*p* < 0.001) across the entire microarray. In addition, the overall range of fluorescence intensity was very similar in repeated experiments, indicating high reproducibility of serum IgG levels based on Luminex bead-based immunoassay detection (Figure 1H). These results indicate that we have successfully established a method for detecting antibody levels after vaccination using Luminex technology.

### 3.3. Longitudinal Observation of Changes in Anti-RBD Antibody Levels after CoronaVac Vaccination

**Figure 2:**
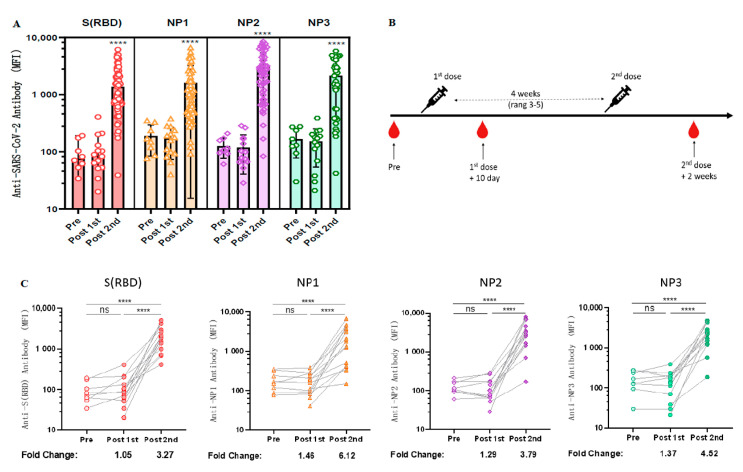
Longitudinal observation of dynamic changes in anti-RBD antibody levels after CoronaVac vaccination. (**A**) Negative sera (*n* = 8), serum samples after the first dose of CoronaVac (*n* = 14), and serum samples after two doses of CoronaVac (*n* = 55) were tested for anti-RBD IgG and anti-NP IgG levels using the Luminex technique. ANOVA was applied to test whether the differences were statistically significant. (**B**) Schematic diagram of the timing of vaccination and peripheral blood sampling. (**C**) Dynamic changes in the antibody immune response to anti-SARS-CoV-2 in serum samples (*n* = 14) from the cohort vaccinated with CoronaVac. MFI values were logged and analyzed for statistical significance using paired *t*-test. **** *p* < 0.0001.

As spike proteins and nucleocapsids have been widely used as antigens for the diagnosis of COVID-19 [23], we next explored the longitudinal antibody responses against these two antigenic proteins. Due to the large molecular weight of NP protein, we wanted to determine whether specific antibodies could be generated against different epitopes of NP protein after vaccination with inactivated COVID-19 vaccine. Therefore, three purchased NP antigen protein fragments [NP1 (AA: 1–180), NP2 (AA: 120–300), NP3 (AA: 240–419)] were coupled to different colored Luminex microspheres, co-incubated with diluted serum, and then PE-labeled secondary antibodies were added and their fluorescence intensity was detected using a Luminex instrument. The results revealed that both RBD and NP elicited significant immune responses in almost all volunteers who received two doses of CoronaVac but only weak signals in the unvaccinated and one-dose-only groups (Figure 2A).

Fourteen volunteers who received CoronaVac were followed up for serological longitudinal monitoring of antibodies to RBD and NP, and peripheral blood was collected before vaccination, within 10 days after the first dose, and 2 weeks after the second dose (Figure 2B). Based on our previous experimental results, the standard curve determined by the gradient dilution of monoclonal antibodies and the measured log MFI values was used to calculate the changes of antibody levels before and after vaccination. The results revealed that the antibody levels of anti-RBD IgG, anti-NP1 IgG, anti-NP2 IgG, and anti-NP3 IgG in serum increased 3.27-fold [96.375 (95% CI 48.47 to 144.28) vs. 2193.25 (95% CI 1356.49 to 3030.01), *p* < 0.0001], 6.12-fold [89.81 (95% CI 71.34 to 178.28) vs. 3599.25 (95% CI 1034.46 to 3364.04), *p* < 0.0001], 3.79-fold [126.25 (95% CI 85.39 to 167.11) vs. 3307.11 (95% CI 1191.78 to 4702.45), *p* < 0.0001], and 4.52-fold [166.50 (95% CI 92.25 to 240.75) vs. 2324.36 (95% CI 1149.41 to 3156.31), *p* < 0.0001], respectively, compared with those before COVID-19 vaccination (Figure 2C).

### 3.4. Cross-Sectional Observation of Anti-SARS-CoV-2 Antibodies Induced by Different Vaccinations

**Figure 3:**
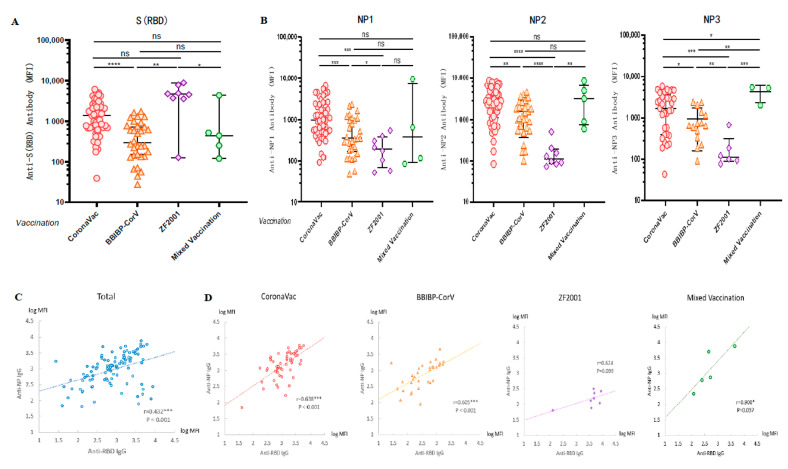
Vaccination with different types of COVID-19 vaccines induced high levels of anti-SARS-CoV-2 antibodies. (**A**,**B**) Cross-sectional observation of IgG levels of anti-RBD and anti-NP produced in vivo after vaccination with different types of vaccines. MFI values were logged and then compared two-by-two for multiple sample means using the LSD (Least-Significant Difference) test. (**C**,**D**) Analysis of the correlation between anti-RBD and anti-NP IgG in sera after vaccination. Normally distributed MFI values were logged, and correlation analysis was performed using Pearson test. * *p* < 0.05, ** *p* < 0.01, *** *p* < 0.001, and **** *p* < 0.0001.

A cross-sectional serological assay was performed on all volunteers to compare the levels of anti-WT-RBD IgG and anti-WT-NP IgG antibodies produced after vaccination with different vaccines. We applied the Luminex technique to detect the antibody levels of anti-RBD IgG in serum and found that the anti-RBD IgG induced in the groups vaccinated with CoronaVac and ZF2001 was higher than that in the group vaccinated with BBIBP-CorV (*p* < 0.0001; *p* = 0.0009) (Figure 3A). The results showed that the induction rates of anti-NP1 (*p* = 0.0003; *p* = 0.0458), anti-NP2 (*p* < 0.0001; *p* < 0.0001), and anti-NP3 IgG (*p* = 0.0002; *p* = 0.004) were significantly lower in the three doses of ZF2001 vaccination group than in the CoronaVac and BBIBP-CorV vaccination groups (Figure 3B), because the vaccine antigen designed by Zhi Fei Biological was the recombinant coronavirus spike glycoprotein receptor binding region NCP-RBD protein. Therefore, the finding that recipients of this vaccine had lower levels of NP antibodies was not surprising. In fact, the MFI values were similar to those observed for pre-vaccinated and COVID-19 negative samples (Figure 1F and Figure 2A).

Since COVID-19 patients have strong antibody responses to both S and N antigen proteins, there was a significant correlation between anti-RBD IgG and anti-NP IgG levels in the serum of patients [23,24]. However, vaccinations with inactivated COVID-19 vaccines (CoronaVac and BBIBP-CorV) were the injection of inactivated intact coronavirus into the human body, so we speculated that there may also be a certain correlation between anti-RBD IgG and anti-NP IgG levels in serum after inactivated vaccination. To investigate the correlation between humoral immune responses induced by various antigenic proteins, we calculated the Pearson correlation coefficients between anti-RBD IgG and anti-NP IgG. We first took the logarithm of the MFI values of anti-NP1, NP2 and NP3 IgG levels to calculate the average value, and then calculated the Pearson correlation coefficient r = 0.432 (*p* < 0.001) between the anti-RBD antibody level and anti-NP antibody level (Figure 3C). A high correlation was observed between the levels of anti-RBD IgG and anti-NP IgG, regardless of whether the vaccination was CoronaVac (r = 0.638, *p* < 0.001), BBIBP-CorV (r = 0.605, *p* < 0.001), or a mixture of two doses of inactivated vaccines (r = 0.900, *p* = 0.037). In contrast, no significant correlation was observed between the levels of anti-RBD IgG and anti-NP IgG induced by the group vaccinated with ZF2001 (r = 0.624, *p* = 0.099) (Figure 3D), because the vaccine antigen designed by Zhi Fei Biological was the recombinant protein RBD of coronavirus and did not contain antigenic proteins of other coronaviruses such as NP. 

### 3.5. Linear Regression Analysis of Factors Related to the Influence of Antibody Level

To investigate the factors associated with the levels of anti-RBD IgG and anti-NP IgG after vaccination, we performed one-way linear regression analysis of seven relevant factors, including sex, blood group, age, BMI, type of vaccination, days between vaccinations, and days between the completion of the last vaccination and the collection of blood samples, with anti-RBD IgG and anti-NP IgG antibody levels.

We found that the different brands of COVID-19 vaccines were closely related to the level of antibodies against each antigenic protein. Anti-RBD IgG levels were significantly higher in ZF2001 than in CoronaVac recipients (b = 0.440, [95% CI 0.017–0.863], *p* = 0.042), possibly because the ZF2001 group received three doses of vaccine while the other vaccination groups received only two doses of vaccine. Regarding the detection of anti-NP IgG levels, we found that the levels of anti-NP IgG after vaccination with BBIBP-CorV and ZF2001 were lower than those in CoronaVac recipients (Table 2).

We also found that age was negatively correlated with anti-NP2 IgG (b = −0.028 [95% CI −0.048–−0.007], *p* = 0.010). In addition, the length of time between the completion of the last dose of vaccination and the collection of blood samples influenced the level of anti-WT-RBD IgG antibody levels (b = −0.003 [95% CI −0.006–0.000], *p* = 0.047) (Table 2). The results of this univariate linear regression showed that the humoral immune response after vaccination was related to the vaccine brand, and the number of days between the last vaccination and blood collection.

### 3.6. Antibodies Induced by Vaccination Can Cross-React with the RBD Antigen of VOCs

**Figure 4:**
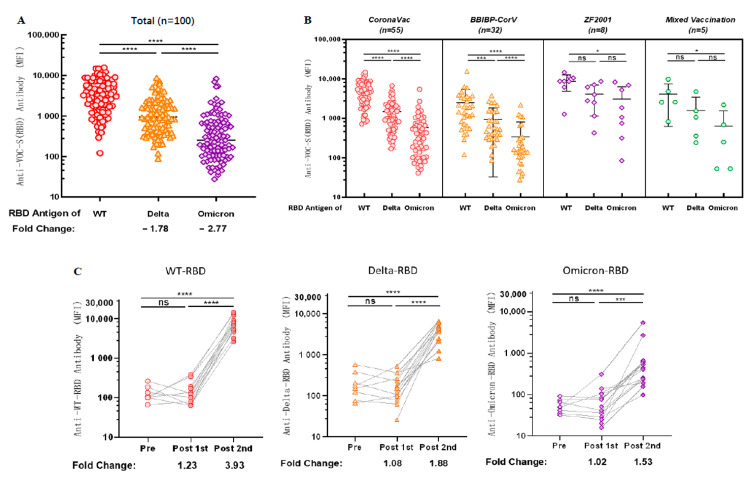
Antibodies induced by vaccination show cross-reactivity with the RBD antigen of VOCs. (**A**) Cross-reactivity of antibodies in sera to RBD antigens of VOCs after vaccination. (**B**) Cross-reactivity of IgG against RBD antigen of VOCs in sera was induced after different types of vaccination. (**A**,**B**) MFI values were logged and then compared two-by-two for multiple samples means using the LSD (Least-Significant Difference) test. (**C**) Dynamic changes of IgG cross-reactivity with RBD antigen of VOCs in serum samples from a cohort vaccinated with CoronaVac. MFI values were logged and analyzed for statistical significance using paired *t*-test. * *p* < 0.05, *** *p* < 0.001, and **** *p* < 0.0001.

Considering the problem of VOCs, we examined the cross-reactivity of antibodies against the RBD antigens of VOCs in sera after vaccination. We observed 1.78-fold [1276.89 (95% CI 951.96 to 1601.83) vs. 3827.55 (95% CI 3079.95 to 4575.15), *p* < 0.0001] and 2.77-fold [612.62 (95% CI 353.80 to 871.45) vs. 3827.55 (95% CI 3079.95 to 4575.15), *p* < 0.0001] decreases in cross-reactivity with Delta-RBD and Omicron-RBD (B.1.1.529), respectively, compared with antibodies against WT-RBD (Figure 4A).

Subsequently, we explored the cross-reactivity of IgG with the RBD antigen of VOCs in sera induced by vaccination with different brands of COVID-19 vaccines; anti-WT-RBD IgG was significantly higher than anti-Delta-RBD IgG (*p* < 0.0001) and anti-Omicron-RBD IgG (*p* < 0.0001) in the group vaccinated with CoronaVac and BBIBP-CorV. In contrast, anti-Omicron-RBD IgG in the sera of both the groups vaccinated with ZF2001 and the group mixed-vaccinated with two different types of inactivated COVID-19 vaccine were lower than anti-WT-RBD IgG (*p* = 0.0226; *p* = 0.0233, respectively) (Figure 4B). Although there is no statistical significance between WT, Delta and Omicron IgG levels for ZF2001 and mixed vaccination, but the trend is similar to what was observed for the other two vaccines. The most likely reason for statistical insignificance is the low number of tested samples.

Cross-reactivity of antibodies against VOCs was dynamically observed using sera from our follow-up longitudinal cohort of 14 CoronaVac-vaccinated volunteers. Anti-WT-RBD IgG, anti-Delta-RBD IgG, and anti-Omicron-RBD IgG levels increased 3.93 times [7513.69 (95% CI 4285.07 to 10742.30) vs. 132.38 (95% CI 79.61 to 185.14), *p* < 0.0001], 1.88 times [4243.88 (95% CI 2332.13 to 6155.62) vs. 220.94 (95% CI 72.64 to 369.23), *p* < 0.0001], and 1.53 times [1259.31 (95% CI 313.58 to 2832.20) vs. 54.88 (95% CI 37.58 to 72.18), *p* < 0.0001], respectively, compared with those before vaccination (Figure 4C), indicating that antibodies produced in the body after two doses of CoronaVac could cross-react with SARS-CoV-2 VOCs.

## 4. Discussion

To analyze the antibody response after vaccination with COVID-19 vaccines, we developed a method to detect antibody levels after vaccination by applying a high-throughput bead-based microarray system in which Luminex antigen microsphere arrays containing three RBD proteins of SARS-CoV-2 (WT, Delta, and Omicron) and WT nucleocapsid NP were used to explore the anti-SARS-CoV-2 antibodies induced after vaccination with different COVID-19 vaccines by means of longitudinal tracking and cross-sectional observation. Our results showed that only weak signals of antibodies were detected in the sera of the unvaccinated and one-dose-only groups and that the levels of specific antibodies increased notably after completion of two doses (CoronaVac or BBIBP-CorV) or three doses (ZF2001) of COVID-19 vaccines. Inactivated vaccination also produced antibodies that could effectively bind multi-antigens to the S and N antigen proteins of SARS-CoV-2. We also observed that antibodies after COVID-19 vaccination could cross-react to some extent with Delta and Omicron variants, suggesting that completion of COVID-19 vaccination (two or three doses) may provide some protection against VOC infection and may also indicate that booster vaccination is essential.

Our longitudinal follow-up of 14 volunteers revealed that vaccination with two doses of CoronaVac elicited a strong antibody response, which was similar to previous findings [25]. We applied Luminex technology to cross-sectionally monitor the antibody levels of anti-WT-RBD IgG and found that the levels of anti-RBD IgG were higher after three doses of ZF2001 than after two doses of BBIBP-CorV, a result consistent with real-world protective power data [7]. We found a significant correlation between anti-RBD IgG and anti-NP IgG levels, as reported in many studies [17,26]. We also performed a linear regression analysis of the relevant factors affecting antibody levels and found that the length of time between the completion of the last dose of vaccination and the collection of blood samples influenced the level of anti-WT-RBD IgG levels, which is consistent with the decrease in titers of serum-neutralizing antibodies over time after BNT162b2 and ChAdOx1 vaccination [27,28,29].

Serological tests are particularly important for exploring the levels of antibodies after vaccination and their protective efficacy [26]. There are different experimental methods for serological testing, including enzyme-linked immunosorbent assay (ELISA), lateral flow immunoassay (LFIA), chemiluminescent immunoassay (CLIA), viral neutralization test (VNT), and pseudotyped viral neutralization assays. VNT is the gold standard of serological tests [30], which allows the direct assessment of the neutralization capacity of antibodies in serum. However, these assays are either time-consuming and experimentally demanding (VNT) or simple and rapid but less sensitive (LFIA) [31] and are not suitable for large-scale serum monitoring [32]. Since these assays have limitations in large-scale serum monitoring, the analysis of serum antibody levels using immune microsphere arrays established by the Luminex system is of great interest. This platform allows for the simultaneous detection of antibodies against different antigens of SARS-CoV-2, which can significantly improve the specificity and sensitivity of serological assays. The immunobead-based assay requires only a lower volume of serum and a lower cost [33]. This method is simple to operate and can quantitatively analyze antibody levels, providing a highly accessible and practical method for antibody detection after COVID-19 vaccination [34,35].

Owing to the extremely high transmissibility and infectivity of Omicron variant (B.1.1.529), breakthrough infections are increasing dramatically, even in areas with high vaccination coverage [19]. Similar to some recently published studies showing decreased antibody titers against Omicron in the sera of COVID-19 vaccine recipients [17,36,37], we observed 1.78-fold and 2.77-fold decreases in cross-reactivity with Delta-RBD and Omicron-RBD, respectively, compared to antibodies against wild-type coronavirus. In a real-world test-negative case-control study, the effectiveness of two doses of inactivated vaccine was 59% for B.1.617.2 [38]. This finding is consistent with that of our study—a significant humoral immune response to the Delta variant occurred after two doses of CoronaVac.

More importantly, regarding the neutralizing activity of the Omicron variant, the neutralizing activity of sera from two-dose BBIBP-CorV against Omicron variant subtypes only reached the minimum detectable limit [39], which is a real concern for the protection against breakthrough B.1.1.529 infection. Therefore, booster vaccination may be useful. There have been studies that have explored changes in serum neutralizing antibodies after a third dose of CoronaVac [40,41,42] and have shown that neutralization titers (GMT) increased rapidly if a booster was administered 6–8 months after the second dose of CoronaVac, suggesting that a third vaccination could significantly induce an immune recall response in specific memory B cells. Overall, the results of other related studies and our study support the promotion of booster vaccination to reduce the rate of COVID-19 infections, hospitalizations, and mortality [17,18,43].

Our study had some limitations. The sample size of the study was relatively small, and we longitudinally followed only 14 participants who received CoronaVac, which was a short follow-up period that did not allow for a comprehensive assessment of the effect of time variation on antibody levels. In future studies, it will be necessary to extend the follow-up period to 6 months or ≥1 year, and the trend of antibody levels over time must be mathematically modeled [44]. Participant age was of concern, as the study only included persons between 19–23 years. The range was very narrow and, thus, evaluation of the effect of age on the measured IgG parameters seemed not very relevant. Furthermore, our study only investigated serum IgG levels, and the protective effect of antibody neutralization induced by vaccination and T-cell responses remains unclear. The Luminex technology is indeed well suited for large-scale evaluation of vaccination campaigns. However, our study lacks a functional validation of the Luminex technique, as this requires a comparison with the gold standard, such as the classical ELISA or virus neutralization assays. Further studies are required to address these issues.

## Figures and Tables

**Table 1:** Demographic characteristics of study subjects with COVID-19 vaccination.

	CoronaVac (*n* = 55)	BBIBP-CorV (*n* = 32)	ZF2001 (*n* = 8)	Mixed Vaccination (*n* = 5)	All Participants (*n* = 100)	*p* Value
**Gender**						0.424
Male	21 (38.18%)	17 (53.13%)	3 (37.50%)	1 (20.00%)	42 (42.00%)	
Female	34 (61.82%)	15 (46.88%)	5 (62.50%)	4 (80.00%)	58 (58.00%)	
**Blood Type**						0.751
A	18 (32.73%)	9 (28.13%)	1 (12.50%)	2 (20.00%)	30 (30.00%)	
B	13 (23.64%)	8 (25.00%)	1 (12.50%)	2 (20.00%)	24 (24.00%)	
AB	1 (1.82%)	1 (3.13%)	0 (0.00%)	0 (0.00%)	2 (2.00%)	
O	20 (36.36%)	14 (43.75%)	6 (75.00%)	1 (20.00%)	41 (41.00%)	
**Age, median age (IQR)**	20 (19–23)	19.5 (19–22.75)	21 (20–24.5)	19 (19–19)	20 (19–23)	0.257
**BMI (Kg/m^2^) (IQR)**	21.34 (19.53–23.72)	20.96 (19.58–24.96)	21.98 (20.96–25.79)	20.96 (20.42–21.77)	21.51 (19.31–24.61)	0.678
**The median interval days between two vaccinations (IQR)**	30 (23–31)	27 (23–34)	The first and second dose (IQR)	27 (21–28)	30 (23–33)	0.735
			32 (30.5–35.5)			
			The second and third dose (IQR)			
			33.5 (30.5–42.5)			
**The median interval days between the last dose and the first blood collection (IQR)**	59 (14–103)	82 (54–117)	88.5 (61–104.5)	80 (76–82)	76 (30.5–109.25)	0.298

Different background colors and words in bold font were used in the table to distinguish the six demographic characteristics collected in the study more clearly.

**Table 2:** Univariate linear regression models of WT-Ab (IgG) immune responses.

Efficient	Anti WT-RBD IgG			Anti WT-NP1 IgG			Anti WT-NP2 IgG			Anti WT-NP3 IgG		
	Estimates	CI (95%)	*p* Value	Estimates	CI (95%)	*p* Value	Estimates	CI (95%)	*p* Value	Estimates	CI (95%)	*p* Value
**Gender**	−0.171	−0.418 to 0.076	0.172	−0.26	−0.511 to −0.009	0.042 *	−0.05	−0.293 to 0.192	0.680	−0.086	−0.450 to 0.279	0.638
**Blood Type**	0.069	−0.015 to 0.153	0.106	−0.003	−0.087 to 0.082	0.956	−0.031	−0.116 to 0.053	0.466	−0.086	−0.193 to 0.056	0.276
**Age**	−0.016	−0.038 to 0.006	0.162	−0.017	−0.039 to 0.005	0.130	−0.028	−0.048 to −0.007	0.010 *	−0.009	−0.047 to 0.028	0.61
**BMI(Kg/m^2^)**	0.004	−0.036 to 0.045	0.834	−0.047	−0.088 to −0.007	0.023 *	−0.032	−0.071 to 0.007	0.108	−0.038	−0.094 to 0.018	0.181
**Vaccination brand**												
CoronaVac												
BBIBP-CorV	−0.513	−0.747 to −0.278	0.000 *	−0.384	−0.638 to −0.131	0.004 *	−0.272	−0.479 to −0.064	0.011 *	−0.337	−0.689 to 0.015	0.060
ZF2001	0.440	0.017 to 0.863	0.042 *	−0.728	−1.162 to −0.294	0.001 *	−1.298	−1.673 to −0.924	0.000 *	−0.977	−1.483 to −0.471	0.000 *
Mixed Injection	−0.228	−0.808 to 0.352	0.436	−0.022	−0.740 to 0.696	0.951	0.143	−0.371 to 0.656	0.582	0.552	−0.504 to 1.608	0.298
**The interval days between** **two vaccinations (IQR)**	0.011	−0.003 to 0.024	0.117	0.004	−0.010 to 0.019	0.546	−0.008	−0.022 to 0.005	0.210	−0.005	−0.026 to 0.015	0.595
**The interval days between** **the last dose andthe first blood collection (IQR)**	−0.003	−0.006 to 0.000	0.047 *	−0.002	−0.005 to 0.001	0.147	−0.002	−0.004 to 0.001	0.276	−0.003	−0.007 to 0.000	0.081

Shown are four univariate linear regression models of WT-Ab (IgG) immune responses. Variables include gender, blood type, age, body mass index (BMI), vaccination brand, the interval days between two vaccinations and the interval days between the second dose and the first blood collection. Reference variable for vaccination brand is CoronaVac. Different background colors and words in bold font were used in the table to distinguish the seven relevant factors more clearly. * Indicates statistical significance.

## Data Availability

The materials, data, and any associated protocols that support the findings of this study are available from the corresponding authors upon request.

## Supplementary Materials

The following supporting information can be downloaded at: https://www.mdpi.com/article/10.3390/vaccines11020442/s1, Figure S1: Expression and purification of soluble antigenic protein RBD; Table S1: Report of the collection time of each sample.

## References

[1] Polack F.P., Thomas S.J., Kitchin N., Absalon J., Gurtman A., Lockhart S., Perez J.L., Pérez Marc G., Moreira E.D., Zerbini C. (2020). Safety and efficacy of the BNT162b2 mRNA COVID-19 vaccine. N. Engl. J. Med..

[2] Baden L.R., El Sahly H.M., Essink B., Kotloff K., Frey S., Novak R., Diemert D., Spector S.A., Rouphael N., Creech C.B. (2021). Efficacy and Safety of the mRNA-1273 SARS-CoV-2 Vaccine. N. Engl. J. Med..

[3] Voysey M., Clemens S.A.C., Madhi S.A., Weckx L.Y., Folegatti P.M., Aley P.K., Angus B., Baillie V.L., Barnabas S.L., Bhorat Q.E. (2021). Safety and efficacy of the ChAdOx1 nCoV-19 vaccine (AZD1222) against SARS-CoV-2: An interim analysis of four randomised controlled trials in Brazil, South Africa, and the UK. Lancet.

[4] Sadoff J., Gray G., Vandebosch A., Cárdenas V., Shukarev G., Grinsztejn B., Goepfert P.A., Truyers C., Fennema H., Spiessens B. (2021). Safety and Efficacy of Single-Dose Ad26.COV2.S Vaccine against Covid-19. N. Engl. J. Med..

[5] Palacios R., Patiño E.G., Piorelli R.D.O., Conde M.T.R.P., Batista A.P., Zeng G., Xin Q., Kallas E.G., Flores J., Ockenhouse C.F. (2020). Double-Blind, Randomized, Placebo-Controlled Phase III Clinical Trial to Evaluate the Efficacy and Safety of treating Healthcare Professionals with the Adsorbed COVID-19 (Inactivated) Vaccine Manufactured by Sinovac – PROFISCOV: A structured summary of a study protocol for a randomised controlled trial. Trials.

[6] Al Kaabi N., Zhang Y., Xia S., Yang Y., Al Qahtani M.M., Abdulrazzaq N., Al Nusair M., Hassany M., Jawad J.S., Abdalla J. (2021). Effect of 2 Inactivated SARS-CoV-2 Vaccines on Symptomatic COVID-19 Infection in Adults. JAMA.

[7] Dai L., Gao L., Tao L., Hadinegoro S.R., Erkin M., Ying Z., He P., Girsang R.T., Vergara H., Akram J. (2022). Efficacy and Safety of the RBD-Dimer–Based Covid-19 Vaccine ZF2001 in Adults. N. Engl. J. Med..

[8] Lan J., Ge J., Yu J., Shan S., Zhou H., Fan S., Zhang Q., Shi X., Wang Q., Zhang L. (2020). Structure of the SARS-CoV-2 spike receptor-binding domain bound to the ACE2 receptor. Nature.

[9] Tian X., Li C., Huang A., Xia S., Lu S., Shi Z., Lu L., Jiang S., Yang Z., Wu Y. (2020). Potent Binding of 2019 Novel Coronavirus Spike Protein by a SARS Coronavirus-Specific Human Monoclonal Antibody. Emerg. Microbes Infect..

[10] Walls A.C., Park Y.-J., Tortorici M.A., Wall A., McGuire A.T., Veesler D. (2020). Structure, Function, and Antigenicity of the SARS-CoV-2 Spike Glycoprotein. Cell.

[11] Andrews N., Stowe J., Kirsebom F., Toffa S., Rickeard T., Gallagher E., Gower C., Kall M., Groves N., O’Connell A.-M. (2022). Covid-19 Vaccine Effectiveness against the Omicron (B.1.1.529) Variant. N. Engl. J. Med..

[12] Chakraborty C., Sharma A.R., Bhattacharya M., Agoramoorthy G., Lee S.-S. (2021). Evolution, Mode of Transmission, and Mutational Landscape of Newly Emerging SARS-CoV-2 Variants. Mbio.

[13] Planas D., Veyer D., Baidaliuk A., Staropoli I., Guivel-Benhassine F., Rajah M.M., Planchais C., Porrot F., Robillard N., Puech J. (2021). Reduced sensitivity of SARS-CoV-2 variant Delta to antibody neutralization. Nature.

[14] Wall E.C., Wu M., Harvey R., Kelly G., Warchal S., Sawyer C., Daniels R., Hobson P., Hatipoglu E., Ngai Y. (2021). Neutralising antibody activity against SARS-CoV-2 VOCs, B.1.617.2 and B.1.351 by BNT162b2 vaccination. Lancet.

[15] Wang P., Casner R.G., Nair M.S., Wang M., Yu J., Cerutti G., Liu L., Kwong P.D., Huang Y., Shapiro L. (2021). Increased resistance of SARS-CoV-2 variant P.1 to antibody neutralization. Cell Host Microbe.

[16] Wang P., Nair M.S., Liu L., Iketani S., Luo Y., Guo Y., Wang M., Yu J., Zhang B., Kwong P.D. (2021). Antibody resistance of SARS-CoV-2 variants B.1.351 and B.1.1.7. Nature.

[17] Zeng C., Evans J.P., Qu P., Faraone J., Zheng Y.-M., Carlin C., Bednash J.S., Zhou T., Lozanski G., Mallampalli R. (2021). Neutralization and Stability of SARS-CoV-2 Omicron Variant. J. BioRxiv.

[18] Riou C., Keeton R., Moyo-Gwete T., Hermanus T., Kgagudi P., Baguma R., Valley-Omar Z., Smith M., Tegally H., Doolabh D. (2022). Escape from recognition of SARS-CoV-2 variant spike epitopes but overall preservation of T cell immunity. Sci. Transl. Med..

[19] Malato J., Ribeiro R.M., Leite P.P., Casaca P., Fernandes E., Antunes C., Fonseca V.R., Gomes M.C., Graca L. (2022). Risk of BA.5 Infection among Persons Exposed to Previous SARS-CoV-2 Variants. N. Engl. J. Med..

[20] Guo J., Guo X., Wang Y., Tian F., Luo W., Zou Y. (2017). Cytokine response to *Hantaan virus* infection in patients with hemorrhagic fever with renal syndrome. J. Med. Virol..

[21] Guo X., Hu J., Luo W., Luo Q., Guo J., Tian F., Ming Y., Zou Y. (2017). Analysis of Sera of Recipients with Allograft Rejection Indicates That Keratin 1 Is the Target of Anti-Endothelial Antibodies. J. Immunol. Res..

[22] Zou Y., Stastny P., Süsal C., Döhler B., Opelz G. (2007). Antibodies against MICA Antigens and Kidney-Transplant Rejection. N. Engl. J. Med..

[23] Jiang H.-W., Li Y., Zhang H.-N., Wang W., Yang X., Qi H., Li H., Men D., Zhou J., Tao S.-C. (2020). SARS-CoV-2 proteome microarray for global profiling of COVID-19 specific IgG and IgM responses. Nat. Commun..

[24] Payne R.P., Longet S., Austin J.A., Skelly D.T., Dejnirattisai W., Adele S., Meardon N., Faustini S., Al-Taei S., Moore S.C. (2021). Immunogenicity of standard and extended dosing intervals of BNT162b2 mRNA vaccine. Cell.

[25] Wang G.-L., Wang Z.-Y., Duan L.-J., Meng Q.-C., Jiang M.-D., Cao J., Yao L., Zhu K.-L., Cao W.-C., Ma M.-J. (2021). Susceptibility of Circulating SARS-CoV-2 Variants to Neutralization. N. Engl. J. Med..

[26] Mariën J., Ceulemans A., Michiels J., Heyndrickx L., Kerkhof K., Foque N., Widdowson M.-A., Mortgat L., Duysburgh E., Desombere I. (2020). Evaluating SARS-CoV-2 spike and nucleocapsid proteins as targets for antibody detection in severe and mild COVID-19 cases using a Luminex bead-based assay. J. Virol. Methods..

[27] Levin E.G., Lustig Y., Cohen C., Fluss R., Indenbaum V., Amit S., Doolman R., Asraf K., Mendelson E., Ziv A. (2021). Waning Immune Humoral Response to BNT162b2 Covid-19 Vaccine over 6 Months. N. Engl. J. Med..

[28] Peng Q., Zhou R., Wang Y., Zhao M., Liu N., Li S., Huang H., Yang D., Au K.-K., Wang H. (2022). Waning immune responses against SARS-CoV-2 variants of concern among vaccinees in Hong Kong. EBioMedicine.

[29] Shrotri M., Navaratnam A.M.D., Nguyen V., Byrne T., Geismar C., Fragaszy E., Beale S., Fong W.L.E., Patel P., Kovar J. (2021). Spike-antibody waning after second dose of BNT162b2 or ChAdOx1. Lancet.

[30] Lassaunière R., Frische A., Harboe Z.B., Nielsen A.C.Y., Fomsgaard A., Krogfelt K.A., Jørgensen C.S. (2020). Evaluation of nine commercial SARS-CoV-2 immunoassays. medRxiv.

[31] Ong D.S., Fragkou P.C., Schweitzer V.A., Chemaly R.F., Moschopoulos C.D., Skevaki C. (2021). How to interpret and use COVID-19 serology and immunology tests. Clin. Microbiol. Infect..

[32] Xiao T., Wang Y., Yuan J., Ye H., Wei L., Liao X., Wang H., Qian S., Wang Z., Liu L. (2021). Early Viral Clearance and Antibody Kinetics of COVID-19 among Asymptomatic Carriers. Front. Med..

[33] Kerkhof K., Canier L., Kim S., Heng S., Sochantha T., Sovannaroth S., Vigan-Womas I., Coosemans M., Sluydts V., Ménard D. (2015). Implementation and application of a multiplex assay to detect malaria-specific antibodies: A promising tool for assessing malaria transmission in Southeast Asian pre-elimination areas. Malar. J..

[34] Dobaño C., Vidal M., Santano R., Jiménez A., Chi J., Barrios D., Ruiz-Olalla G., Melero N.R., Carolis C., Parras D. (2021). Highly Sensitive and Specific Multiplex Antibody Assays to Quantify Immunoglobulins M, A, and G against SARS-CoV-2 Antigens. J. Clin. Microbiol..

[35] Rosado J., Pelleau S., Cockram C., Merkling S.H., Nekkab N., Demeret C., Meola A., Kerneis S., Terrier B., Fafi-Kremer S. (2020). Multiplex assays for the identification of serological signatures of SARS-CoV-2 infection: An antibody-based diagnostic and machine learning study. Lancet Microbe.

[36] Dejnirattisai W., Huo J., Zhou D., Zahradník J., Supasa P., Liu C., Duyvesteyn H.M., Ginn H.M., Mentzer A.J., Tuekprakhon A. (2022). SARS-CoV-2 Omicron-B.1.1.529 leads to widespread escape from neutralizing antibody responses. Cell.

[37] Garcia-Beltran W.F., St Denis K.J., Hoelzemer A., Lam E.C., Nitido A.D., Sheehan M.L., Berrios C., Ofoman O., Chang C.C., Hauser B.M. (2022). mRNA-based COVID-19 vaccine boosters induce neutralizing immunity against SARS-CoV-2 Omicron variant. Cell.

[38] Li X.-N., Huang Y., Wang W., Jing Q.-L., Zhang C.-H., Qin P.-Z., Guan W.-J., Gan L., Li Y.-L., Liu W.-H. (2021). Effectiveness of inactivated SARS-CoV-2 vaccines against the Delta variant infection in Guangzhou: A test-negative case–control real-world study. Emerg. Microbes Infect..

[39] Yao L., Zhu K.-L., Jiang X.-L., Wang X.-J., Zhan B.-D., Gao H.-X., Geng X.-Y., Duan L.-J., Dai E.-H., Ma M.-J. (2022). Omicron subvariants escape antibodies elicited by vaccination and BA.2.2 infection. Lancet Infect. Dis..

[40] Zeng G., Wu Q., Pan H., Li M., Yang J., Wang L., Wu Z., Jiang D., Deng X., Chu K. (2021). Immunogenicity and safety of a third dose of CoronaVac, and immune persistence of a two-dose schedule, in healthy adults: Interim results from two single-centre, double-blind, randomised, placebo-controlled phase 2 clinical trials. Lancet Infect. Dis..

[41] Fiorino F., Ciabattini A., Sicuranza A., Pastore G., Santoni A., Simoncelli M., Polvere J., Galimberti S., Baratè C., Sammartano V. (2022). The third dose of mRNA SARS-CoV-2 vaccines enhances the spike-specific antibody and memory B cell response in myelofibrosis patients. Front. Immunol..

[42] Ciabattini A., Pastore G., Fiorino F., Polvere J., Lucchesi S., Pettini E., Auddino S., Rancan I., Durante M., Miscia M. (2021). Evidence of SARS-CoV-2-Specific Memory B Cells Six Months after Vaccination with the BNT162b2 mRNA Vaccine. Front. Immunol..

[43] Gruell H., Vanshylla K., Tober-Lau P., Hillus D., Schommers P., Lehmann C., Kurth F., Sander L.E., Klein F. (2022). mRNA booster immunization elicits potent neutralizing serum activity against the SARS-CoV-2 Omicron variant. Nat. Med..

[44] Hens N., Ghebretinsae A.H., Hardt K., Van Damme P., Van Herck K. (2014). Model based estimates of long-term persistence of inactivated hepatitis A vaccine-induced antibodies in adults. Vaccine.

