# Supplementary material for: Analysis of Antibodies Induced after SARS-CoV-2 Vaccination Using Antigen Coded Bead Array Luminex Technology
Source: Vaccines (Basel). 2023 Feb 15;11(2):442. doi: 10.3390/vaccines11020442 (PMC9964277; doi:10.3390/vaccines11020442)
Supplement: Supplementary file 1 [file vaccines-11-00442-s001.zip › Figure S1-Uncropped Blots.pdf]

Figure S1.

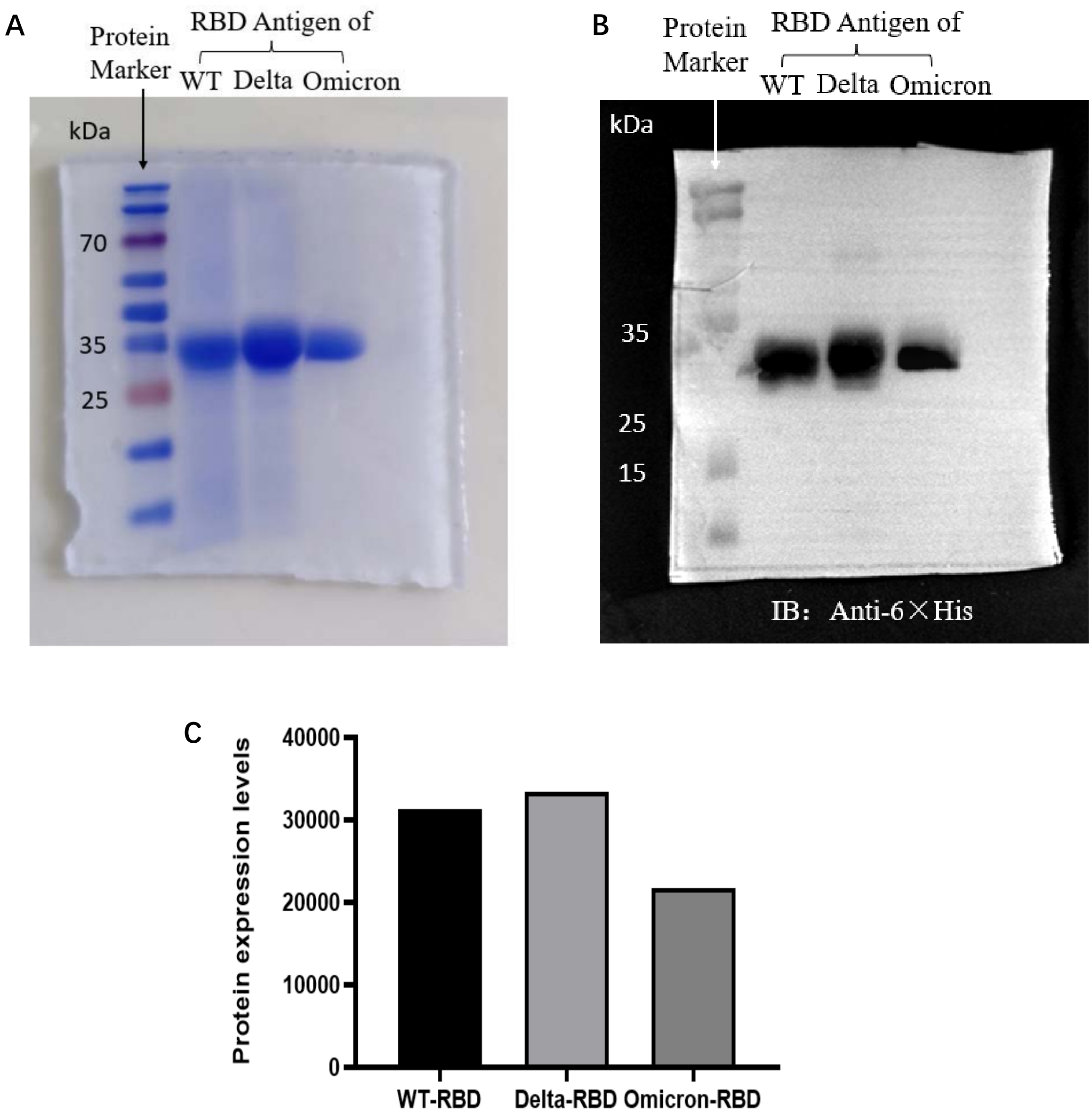

Figure S1. **Expression and purification of soluble antigenic protein RBD. (A)** The report of Coomassie brilliant blue staining gel, original, uncropped and unadjusted images. **(B)** The report of western blot, original, uncropped and unadjusted images. **(C)** The report of Western Blot gray values. **(A, B, C)** Purchased Omicron-RBD antigen protein was used as a control.
